# Peripheral Ameloblastoma Resembling an Exophytic Gingival Lesion: A Report of a Rare Case

**DOI:** 10.7759/cureus.74004

**Published:** 2024-11-19

**Authors:** Abira Chattopadhyay, Arif Hossain, Aritra Chatterjee, Nayana De, Soumen Mandal

**Affiliations:** 1 Oral and Maxillofacial Surgery, Dr. R. Ahmed Dental College and Hospital, Kolkata, IND; 2 Oral and Maxillofacial Surgery, Burdwan Dental College and Hospital, Burdwan, IND

**Keywords:** ameloblastoma, exophytic, gingival, peripheral, recurrence

## Abstract

Peripheral ameloblastoma is a rare, benign, slow-growing odontogenic neoplasm prevalent in the mandible. It originates from the odontogenic epithelium and its remnants, and its histological characteristics are identical to those of intraosseous ameloblastoma. It is less aggressive and invasive than its intraosseous variety, with a low recurrence rate.

This report vividly portrays a rare case of peripheral ameloblastoma in a young male patient, detailing its clinical and surgical presentation. It effectively underscores the importance of considering peripheral ameloblastoma in the differential diagnosis of exophytic gingival lesions.

## Introduction

Ameloblastoma accounts for 1% of all oral and 11% of all odontogenic tumors [1,2]. Ameloblastoma is clinically benign, slow-enlarging, and a painless mass but locally aggressive [3]. WHO classified ameloblastoma into three categories based on clinical-radiological findings [4]: (i) intraosseous multicystic or solid type, (ii) intraosseous unicystic type, and (iii) peripheral or extra-osseous type [4].

Peripheral ameloblastomas are rare tumors, accounting for only 1% to 5% of all ameloblastomas [2,5]. The lesion is primarily found in the mandible (70.9% vs. 29.1% in the maxilla) and most frequently in the gingival margin area [2,5,6].

The histological origin of peripheral ameloblastoma is suggested to derive from the "Serre's pearls," which are the extraosseous cellular residuals of dental lamina epithelium and its remnants in gingiva [1]. It does not invade the underlying bone. However, occasional mild erosion of cortical bone may be seen without invading the bone marrow [1,7]. Radiologically, intrabony invasion is rare; however, peripheral cupping is present due to pressure resorption [7].

Clinically and radiologically, the differential diagnoses of peripheral ameloblastoma are pyogenic granuloma, peripheral giant cell granuloma, and other odontogenic gingival epithelial hamartomas [1,7]. Histologically, it resembles basal cell carcinoma (BCC), the distinction being the pattern of positivity for cytokeratin [1]. As it is a rare entity, the lack of definite guidelines for surgical management presents an intriguing challenge. The lack of infiltration distinguishes the pathology from its intraosseous variety, and the recurrence rate is low. Surgical excision or extensive treatment with a disease-free margin has been advocated [3,7,8].

This article will present a case of peripheral ameloblastoma in the lower alveolar crest at the left-sided canine premolar area, successfully treated surgically. The patient's nine-month follow-up showed no recurrence, confirming the effectiveness of the chosen approach.

## Case presentation

A 17-year-old male patient reported to the Department of Oral and Maxillofacial Surgery, Dr. R. Ahmed Dental College and Hospital, Kolkata, India, with a chief complaint of a painless, slow-growing swelling in the interdental region of the lower left canine and first premolar for the last six to seven months. Notably, there was no history of trauma, infection, or pus discharge related to the lesion. Equally reassuring, there was no systemic disease or significant drug history. The patient was a high school student with no history of smoking and other oral habits.

**Figure 1:**
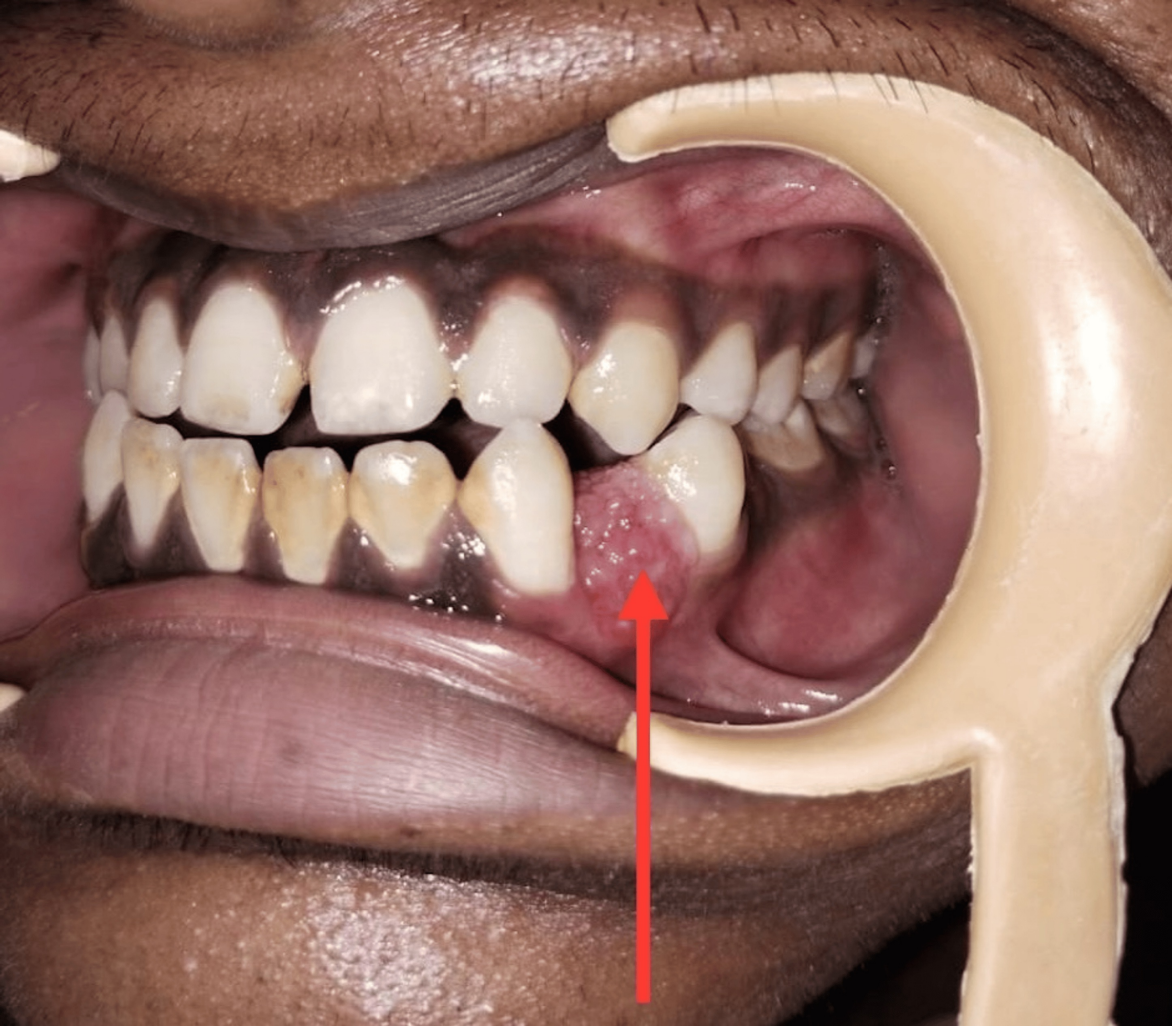
Intraoral image showing lesion

Intraoral examination revealed the presence of a well-circumscribed, spherical, non-tender, and sessile growth with an irregular or granular surface extending from the mandibular left canine to the first premolar region and measuring 1.0 × 1.5 cm in diameter (Figure 1).

**Figure 2:**
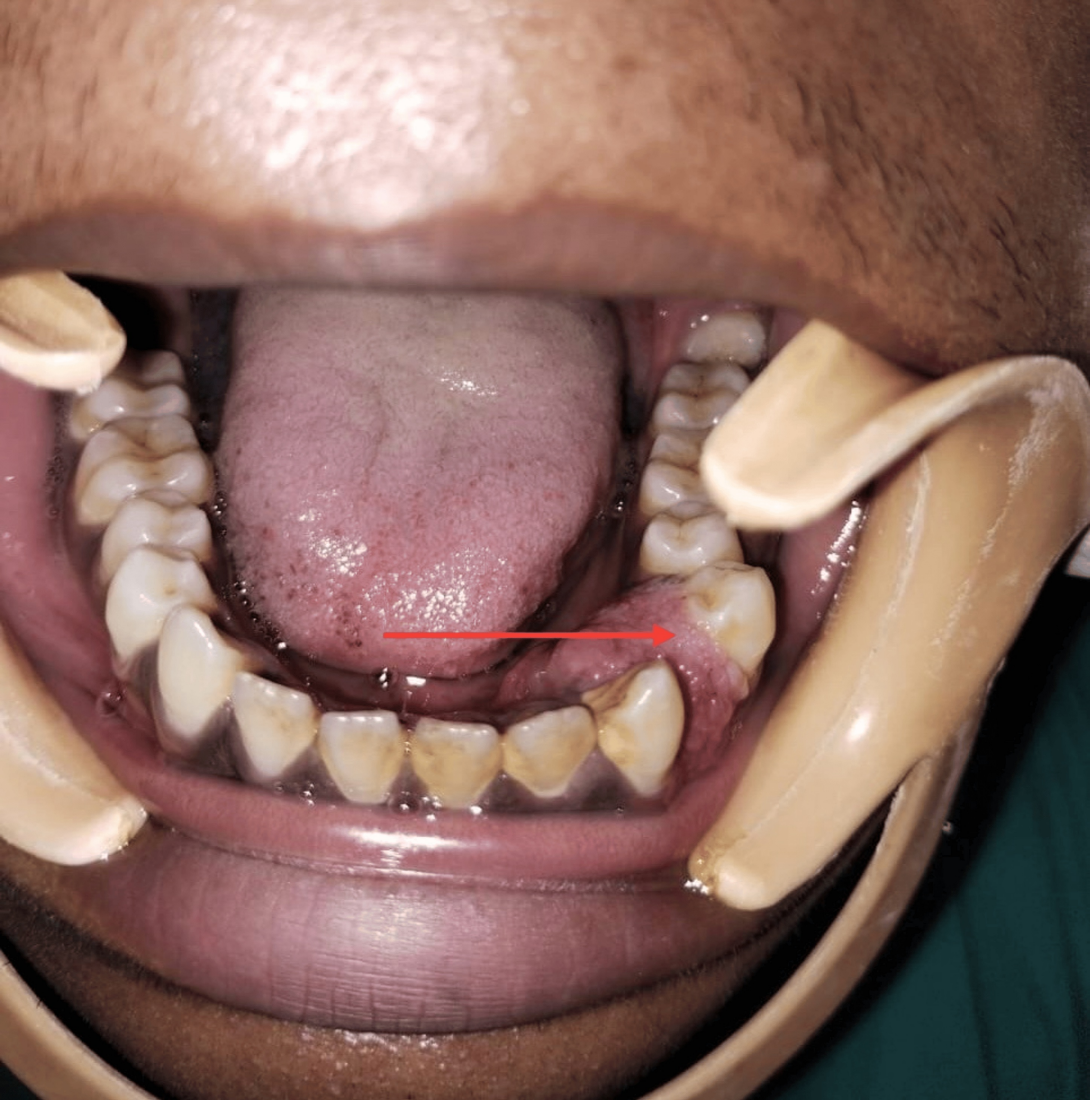
Intraoral photograph Distal to the lesion shows buccoverted mandibular first pre-molar.

The color of the mucosa overlying the swelling was almost the same or slightly reddish compared to the adjacent mucosa. The first premolar was slightly buccoverted (Figure 2).

**Figure 3:**
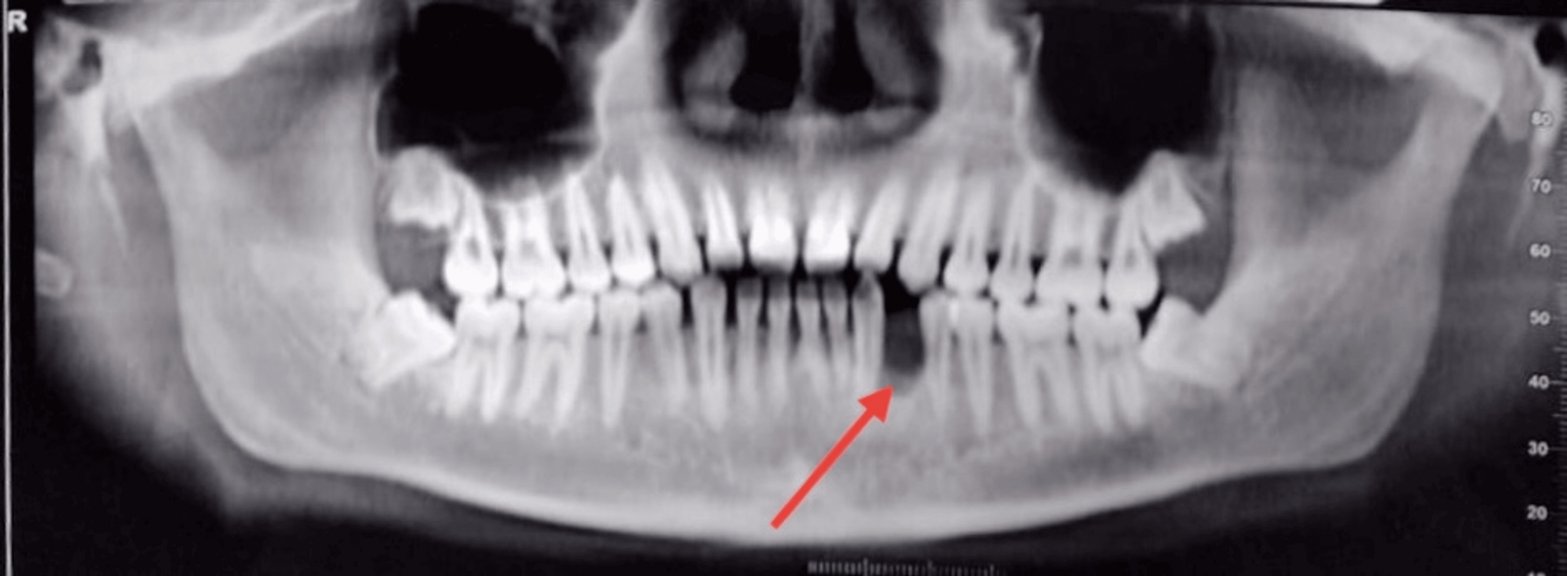
OPG showing bone loss OPG shows pressure resorption of alveolar bone between the mandibular left canine and pre-molar area. OPG: orthopantomogram

There was a periodontal pocket on the distal surface of the canine and the proximal surface of the first premolar. There was no bleeding on probing, and the involved teeth were vital. Orthopantomogram (OPG) showed horizontal bone loss simulating peripheral cupping of alveolar bone between the mandibular left canine and the first premolar region extending up to the apical one-third of the distal surface of the canine (Figure 3).

**Figure 4:**
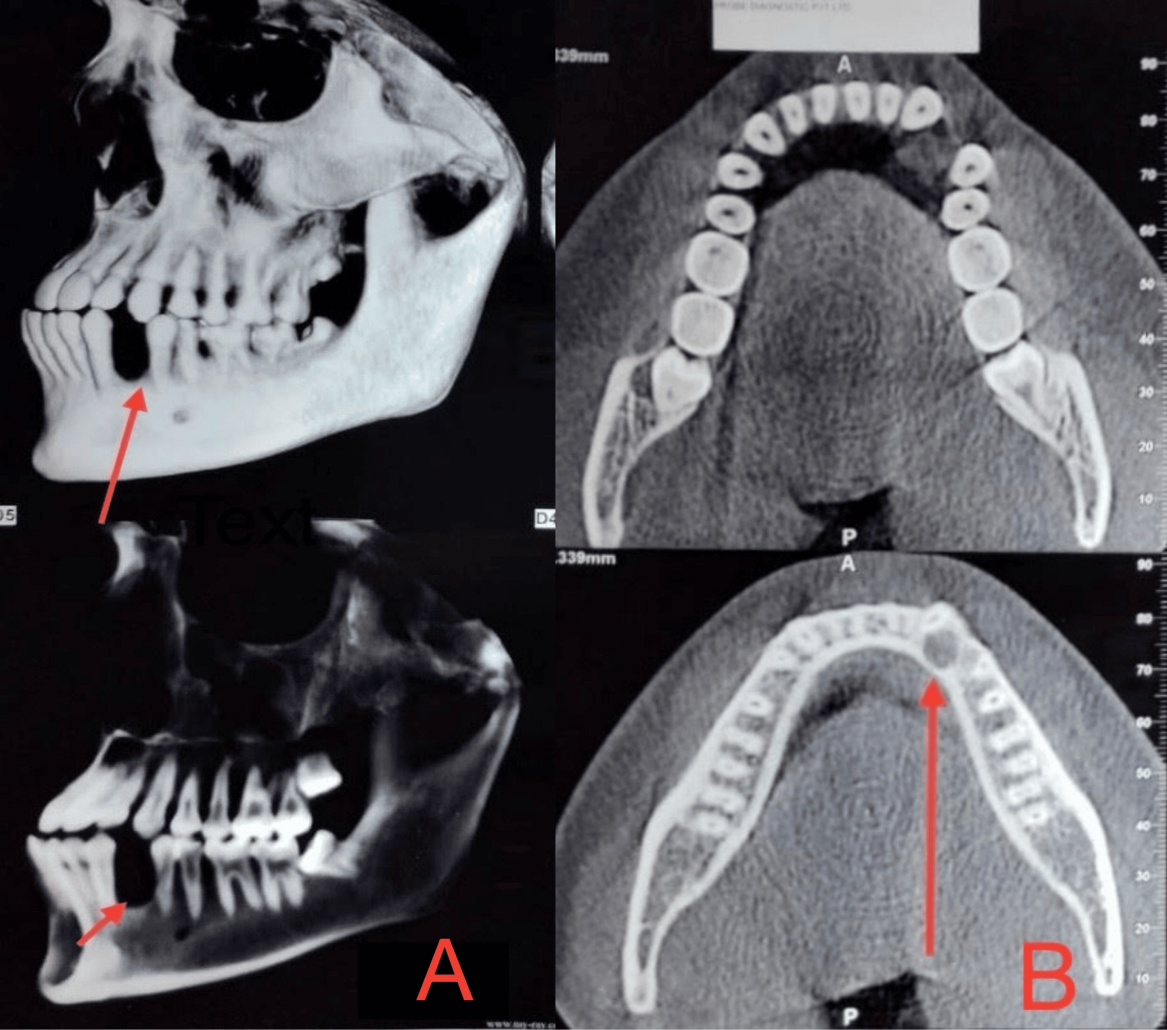
CBCT showing bone loss pattern (A and B) Saucerization or peripheral cupping of the dentoalveolar segment without affecting the basal bone. CBCT: cone-beam computed tomography

CBCT also showed resorption of the alveolar bone (Figures 4A-4B).

**Figure 5:**
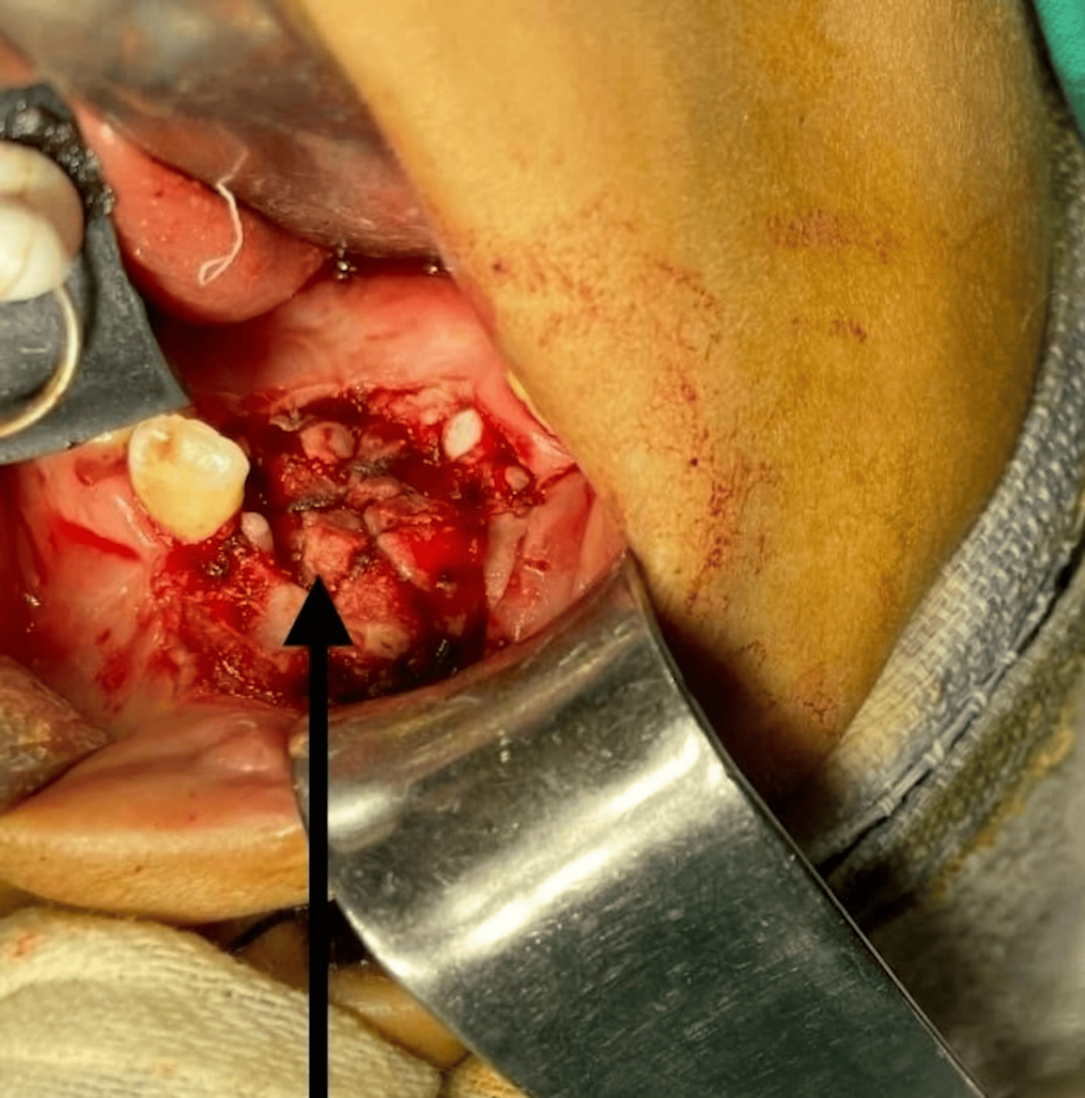
Preoperative image showing peripheral ostectomy Excision of the lesion was done with the extraction of the mandibular left canine and pre-molar.

The clinical diagnosis was peripheral giant cell granuloma, and an excisional biopsy with the extraction of regional teeth was planned. Due to repeated syncopal attacks at the dental chair, surgery was planned under general anesthesia after proper evaluation and consultation with an anesthesiologist. After the soft tissue mass excision, the mandibular left canine and first premolar were extracted, and a vulcanite bur was used for peripheral ostectomy (Figure 5).

**Figure 6:**
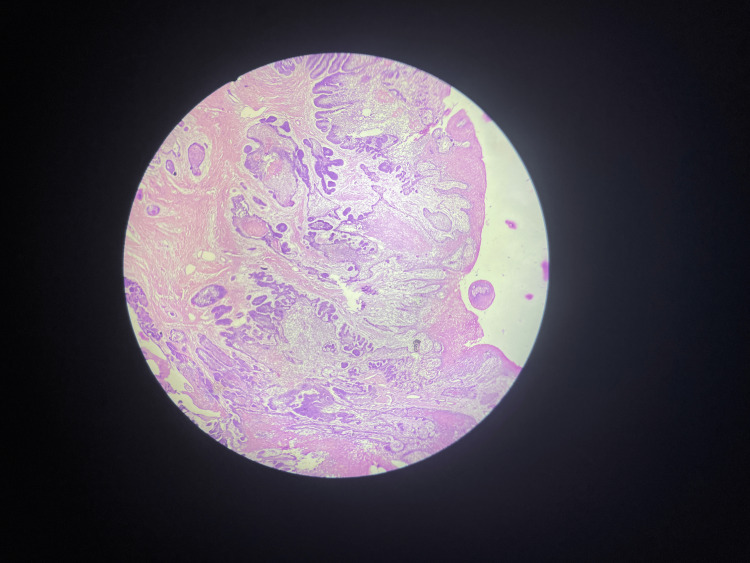
Photomicrograph of the tumor The H&E stained section shows a parakeratinised surface epithelium with underlying odontogenic epithelial cells in the islands. H&E: hematoxylin and eosin

The gross specimen was a single bit of tissue measuring 1.2 × 1.0 cm. The specimen was stained in hematoxylin and eosin and seen under 40x magnification. The photomicrograph of areas exhibiting features consistent with peripheral ameloblastoma originating from a surface epithelium and in the connective tissue stroma, the presence of the tumor islands consisting of a central mass of loosely connected stellate reticulum-like cells surrounded by a layer of palisaded columnar cells with reversely polarized nuclei noted (Figure 6).

**Figure 7:**
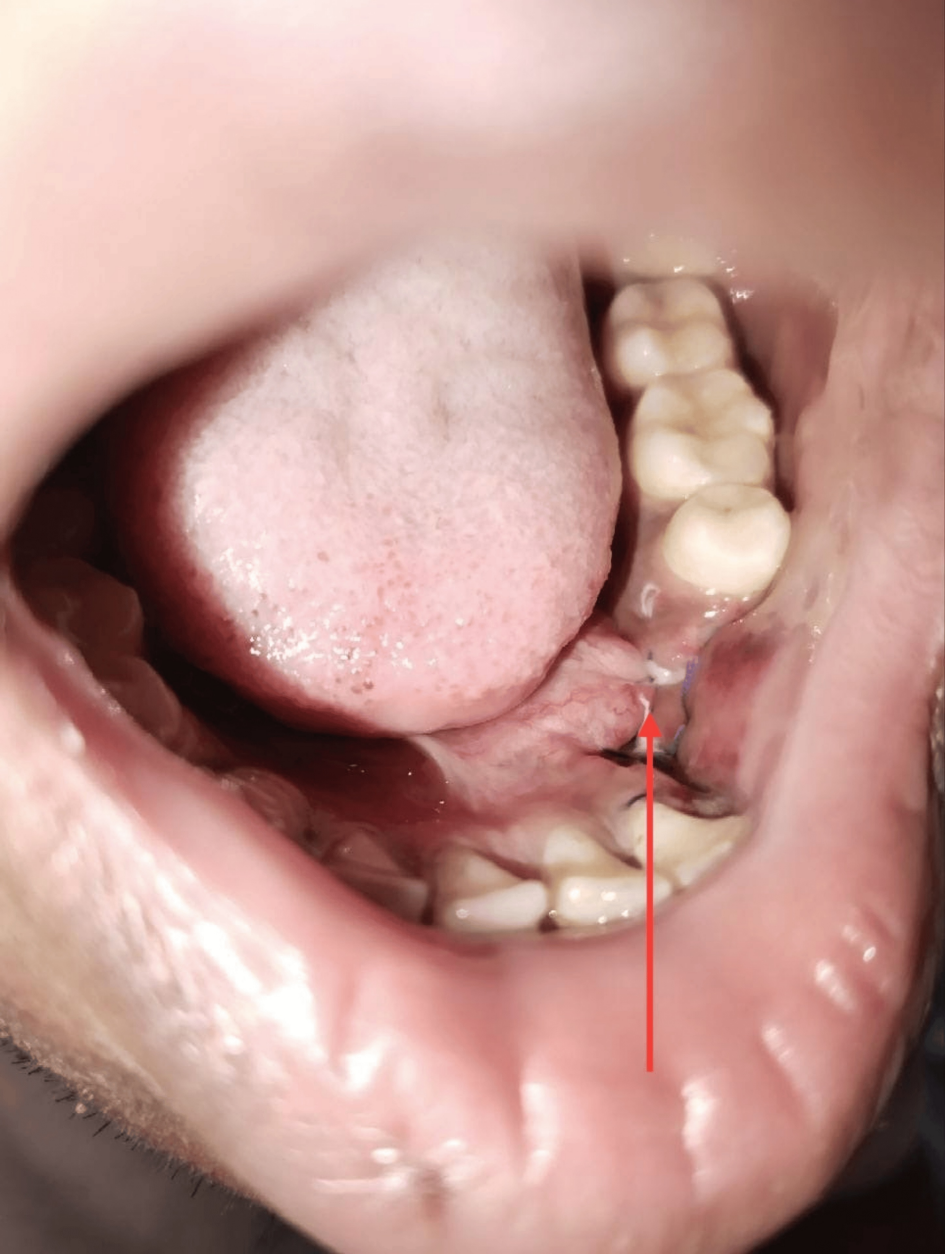
Postoperative image after 10 days The postoperative photograph shows satisfactory healing at the surgical site.

Postoperative healing was uneventful, with no recurrence after a nine-month follow-up (Figure 7).

## Discussion

Peripheral ameloblastoma is a benign, slow-growing odontogenic tumor arising from the odontogenic epithelium [9].

Kuru first described the term "peripheral ameloblastoma" in 1911 [10], but Stanley and Krogh gave a detailed overview in 1959 [11].

The incidence of peripheral ameloblastoma is at a higher age than intraosseous ameloblastoma. 1.9:1 is the male/female ratio compared to 1.2:1 for the intraosseous variety. According to the site, the maxilla/mandible ratio is 1:2.6 [12]. The most common site is the mandibular premolar region (32.6%), followed by the anterior mandible and maxillary tuberosity area. The buccal mucosa, pterygomandibular area, the floor of the mouth, and the base of the tongue are unusual sites affected by peripheral ameloblastoma [1,12].

Most of the time, the lesion clinically resembles gingival lesions with a smooth surface that sometimes may be described as "granular," "pebbly," "papillary," or "warty." The surface color varies between "normal" or "pink" to "red" or "dark red" [12-13]. Masticatory trauma or localized infection may contribute to the reddish color surface ulceration or keratotic surface changes of the lesion. In this reported case, occlusal trauma contributed to the mild reddish color of the lesion.

The histopathogenesis of peripheral ameloblastoma has been extensively discussed. The most probable sources of this lesion are remnants of the dental lamina, the so-called "glands of Serres," odontogenic remnants of the vestibular lamina, pluripotent cells in the basal cell layer of the mucosal epithelium, and pluripotent cells from minor salivary glands [12,14-16]. As different studies have explained, the ultrastructural features include three zones: the deep tumor islands, the "altered" surface epithelium case overlying the tumor, and the area that is transitional between the "normal" and "altered" covering epithelium. These features were similar to those of the central or intraosseous variety [15].

The differential diagnosis of peripheral ameloblastoma: i) pyogenic granuloma, ii) peripheral giant cell granuloma, iii) peripheral ossifying fibroma, iv) papilloma, v) fibroma, vi) peripheral odontogenic fibroma, vii) Baden's odontogenic gingival epithelial hamartoma, as well as viii) BCC [12]. On immunohistochemical analysis, AE1/AE3, KL1, E12, and MNF116 cytokeratin, as seen in human enamel, are positive in peripheral ameloblastoma [14-16]. Peripheral ameloblastoma is positive for cytokeratin 19, which can be differentiated from BCC [1]. Immunohistochemistry is essential as peripheral ameloblastoma and BCC treatment modalities differ and require radicality [1].

Peripheral ameloblastoma, reassuringly, has no invasive potential, and malignant transformation is rare. Though reported, metastasis is extremely rare, providing further reassurance about the condition's severity [5].

The possible effective barrier may be the dense fibrous tissue of the gingiva, periosteum, and alveolar bone to prevent the infiltration of peripheral ameloblastoma [17]. The current treatment modality of choice is a conservative surgical approach, such as supra periosteal surgical excision with adequate disease-free margins [12]. The reported overall recurrence rate ranges from 9% to 20% for supraperiosteal excision. Recurrence may be presented from two months to seven years, with the cause of the quicker recurrences being explained as incomplete surgical excision [2,12]. In our case, a complete surgical excision was done, and the patient has been on regular follow-up for the last eight months with no recurrence noted so far.

## Conclusions

Peripheral ameloblastoma is an uncommon and rare odontogenic tumor that is slow-growing but non-invasive. The common site of the lesion is the gingival tissue of dentulous areas of the jaw; the tumor mimics a typical gingival lesion, such as a fibroma or a pyogenic granuloma, which makes the clinical diagnosis very challenging. Careful histopathological evaluation is essential to diagnose the case. Immunohistochemistry may be helpful for definitive diagnosis. Meticulous excision of the lesion with histopathological evaluation ensures the tumor's margins are clear. Although recurrence is rare, it has been reported in a few cases; treated cases should be followed up for longer to detect local recurrence. Since these lesions are clinically similar to exophytic gingival lesions, peripheral ameloblastoma should be considered in differential diagnosis, and an accurate diagnosis will help to provide better treatment.

## References

[1] Bertossi D, Favero V, Albanese M (2014). Peripheral ameloblastoma of the upper gingiva: report of a case and literature review. J Clin Exp Dent.

[2] Anpalagan A, Tzortzis A, Twigg J, Wotherspoon R, Chengot P, Kanatas A (2021). Current practice in the management of peripheral ameloblastoma: a structured review. Br J Oral Maxillofac Surg.

[3] Zhang X, Tian X, Hu Y, Zhang C, Wei C, Yang X (2018). Oral peripheral ameloblastoma: a retrospective series study of 25 cases. Med Oral Patol Oral Cir Bucal.

[4] El-Naggar AK, Chan JK, Takata T, Grandis JR, Slootweg PJ (2017). The fourth edition of the head and neck World Health Organization blue book: editors' perspectives. Hum Pathol.

[5] Goda H, Nakashiro K, Ogawa I, Takata T, Hamakawa H (2015). Peripheral ameloblastoma with histologically low-grade malignant features of the buccal mucosa: a case report with immunohistochemical study and genetic analysis. Int J Clin Exp Pathol.

[6] On D, Kang M, Ryu J (2019). Peripheral ameloblastoma of the pterygomandibular space: a case report. J Oral Maxillofac Surg Med Pathol.

[7] Vanoven BJ, Parker NP, Petruzzelli GJ (2008). Peripheral ameloblastoma of the maxilla: a case report and literature review. Am J Otolaryngol.

[8] Borrello R, Bettio E, Bacci C, Valente M, Sivolella S, Mazzoleni S, Berengo M (2016). A conservative approach to a peripheral ameloblastoma. Case Rep Dent.

[9] Vezhavendhan N, Vidyalakshmi S, Muthukumaran R, Santhadevy A, Sivaramakrishnan M, Gayathri C (2020). Peripheral ameloblastoma of the gingiva. Autops Case Rep.

[10] Kuru H (1911). About the adamantinoma (Article in German). Zentralbl Allg Pathol.

[11] Stanley H, Krogh H (1959). Peripheral ameloblastoma; report of a case. Oral Surg Oral Med Oral Pathol.

[12] Philipsen HP, Reichart PA, Nikai H, Takata T, Kudo Y. (2001). Peripheral ameloblastoma: biological profile based on 160 cases from the literature. Oral Oncol [Internet].

[13] Pekiner FN, Ozbayrak S, Sener BC, Olgaç V, Sinanoğlu A (2007). Peripheral ameloblastoma: a case report. Dentomaxillofac Radiol.

[14] Isomura ET, Okura M, Ishimoto S, Yamada C, Ono Y, Kishino M, Kogo M (2009). Case report of extragingival peripheral ameloblastoma in buccal mucosa. Oral Surg Oral Med Oral Pathol Oral Radiol Endod.

[15] Gould AR, Farman AG, DeJean EK, Van Arsdall LR (1982). Peripheral ameloblastoma: an ultrastructural analysis. J Oral Pathol.

[16] Yamanishi T, Ando S, Aikawa T (2007). A case of extragingival peripheral ameloblastoma in the buccal mucosa. J Oral Pathol Med.

[17] Gardner DG (1996). Some current concepts on the pathology of ameloblastomas. Oral Surg Oral Med Oral Pathol Oral Radiol Endod [Internet].

